# Piperonylpiperazine Targets RhlI to Reduce Virulence and Potentiate EDTA Sensitivity in *Pseudomonas aeruginosa*

**DOI:** 10.3390/foods15152660

**Published:** 2026-07-29

**Authors:** Jin-Wei Zhou, Yu-Xin Qiu, Hai-Yan Wang, Meng Chen, Jia-Wen Li, Gu-Yitian Xu, Jia-Cheng Liu, Xin-Yu Wu, Yao Zou, Ying Wang, Xiaojuan Tan, Xin-Yu Qian, Jin-Fang Zhou

**Affiliations:** 1 School of Food and Biological Engineering, Xuzhou University of Technology, Xuzhou 221018, China; 2 Anhui Provincial Key Laboratory of Molecular Enzymology and Mechanism of Major Diseases, College of Life Sciences, Anhui Normal University, Wuhu 241000, China; 3 Shaanxi Key Laboratory of Agricultural and Environmental Microbiology, College of Life Sciences, Northwest A&F University, Xianyang 712100, China

**Keywords:** *Pseudomonas aeruginosa*, RhlI protein, quorum sensing, biofilm

## Abstract

*Pseudomonas aeruginosa* is a predominant spoilage organism in meat products. The extensive use of antimicrobial preservatives has, however, led to the emergence of resistant strains. Mitigating the resistance of foodborne *P. aeruginosa* to conventional preservatives has become a critical challenge in food safety. Piperonylpiperazine (Pip), a piperazine derivative sourced from *Piper nigrum*, was first examined in this work as a novel agent capable of both suppressing virulence and enhancing preservative efficacy against *P. aeruginosa*, with its mode of action elucidated. At sub-inhibitory concentrations, Pip strongly suppressed the production of virulence factors and potentiated the susceptibility of *P. aeruginosa* to the common preservative EDTA. Mechanistically, a multi-pronged approach involving pull-down assay, transcriptomic profiling, isothermal titration calorimetry (ITC) analysis, and gene knockout models demonstrated that Pip binds specifically to the LYS-164 and ASP-35 residues of the RhlI synthase, blocking the quorum sensing (QS) signaling cascade. This QS disruption led to reduced virulence factor production and attenuated pathogenicity in a *Caenorhabditis elegans* model. The compromised QS system subsequently induced oxidative stress, which disrupted cell membrane integrity and permeability, thereby potentiating EDTA’s antibacterial action. These findings suggest that Pip is a promising natural additive that can be used in combination with existing preservatives to enhance food safety.

## 1. Introduction

Microbial spoilage poses a significant threat to global food safety, being a primary cause of food deterioration [1]. These microorganisms induce spoilage through manifestations such as viscosity, discoloration, and putrefaction, with the detrimental consequences of potential food intoxication and foodborne diseases, severely impacting product quality and human health [2]. *Pseudomonas aeruginosa* stands as one of the most notorious spoilage microorganisms implicated in both food biodeterioration and foodborne illness [3]. This organism drives spoilage through the action of secreted virulence factors like proteases and elastase, which initiate the deteriorative processes responsible for unacceptable sensory changes such as sliminess and putrefaction [4]. These activities not only render food unpalatable but also pose a substantial risk for foodborne diseases, thereby representing a dual threat to both food industry and public health [5]. The application of antimicrobial preservatives remains one of the principal strategies to control spoilage caused by *P. aeruginosa*. However, the heavy reliance on antimicrobial preservatives has fueled the alarming global spread of *P. aeruginosa* strains exhibiting multidrug-resistant phenotypes [6]. Reversing this trend and resensitizing this strain to antimicrobial interventions now constitutes a pressing challenge and an imperative for ensuring global food safety. In this context, the discovery of quorum sensing (QS) presents a compelling alternative therapeutic route, aiming to disrupt bacterial communication and thereby overcome resistance mechanisms [7,8].

The pathogen *P. aeruginosa* utilizes an intricate quorum sensing circuit-*las*, *rhl*, and *pqs*-which generates the autoinducers of 3-oxo-C12-homoserine lactone (3-oxo-C12-HSL), C4-homoserine lactone (C4-HSL), and *Pseudomonas* Quinolone Signal (PQS), respectively [9,10]. This network is not parallel but a tightly regulated hierarchy with the Las system at the apex, exercising direct transcriptional control over the *rhl* and *pqs* regulons. Since this integrated system globally regulates phenotypes critical for pathogenesis and spoilage-including virulence factor secretion, resistance mechanisms, and infectivity-the development of quorum quenching agents to block this signaling has emerged as a promising anti-virulence strategy [11].

Numerous synthetic compounds have been reported to interfere with the QS circuitry of *P. aeruginosa* [12,13]. However, their cytotoxic effects on mammalian cells restrict broader applications in the food industry [14]. Consequently, there is a growing interest in identifying novel, potent, and low-toxicity QS inhibitors (QSIs) from natural sources [14]. Combining such natural QSIs with conventional antimicrobial agents represents a promising strategy to combat spoilage bacteria and address antimicrobial resistance [15,16]. Ethylenediaminetetraacetic acid (EDTA) is a well-established, cost-effective aminopolycarboxylic acid known for its metal-chelating properties. Approved by the FDA as a food additive to inhibit oxidation in meat products, EDTA also demonstrates antimicrobial activity [17]. Nevertheless, its intrinsic antimicrobial effect is mild, leading to its primary application as an adjuvant or synergist to enhance the efficacy of other antimicrobial agents within the medical and food industries [18]. Hence, the exploration and development of natural preservatives to significantly boost the antibacterial action of EDTA represents a vital area of research. Piperonylpiperazine (Pip), a prominent bioactive component derived from the common seasoning *Piper nigrum*, has traditionally been utilized for its neuroprotective properties [19,20]. However, the putative QS inhibitory activity of Pip has not yet been elucidated, nor has its capacity to potentiate the antibacterial activity of EDTA been ascertained. Consequently, this study was undertaken to evaluate, for the first time, the virulence-attenuating and synergistic activity of Pip against *P. aeruginosa*. The mechanisms underlying this activity are also explored in depth, with the goal of providing a theoretical basis for the management of antimicrobial resistance in *P. aeruginosa*.

## 2. Materials and Methods

### 2.1. Minimum Inhibitory Concentration (MIC) and Growth Profile

We chose *P. aeruginosa* PAO1 but not PA14 for this experiment: PAO1 has a clarified genetic profile and consistent phenotypes suitable as a standard model for food preservative screening, while PA14, a hypervirulent clinical strain, displays unstable phenotypes under bacteriostatic treatment and is typically used for clinical infection research. Edetate disodium (EDTA, purity > 99%) and Pip (purity > 97%) were purchased from Shanghai Yuanye Biotech. The MIC of EDTA and Pip was determined using broth microdilution method with an inoculation of 5 × 10^5^ CFU/mL. Bacterial growth kinetics were determined by inoculating *P. aeruginosa* PAO1 cultures into Luria-Bertani (LB) medium containing increasing concentrations of Pip. After cultivation at 37 °C at 180 rpm for 24 h, cell growth was quantified by measuring the optical density (OD) at 600 nm [21,22].

### 2.2. Anti-Virulence Assay

To assess the secreted protease activity, the sterile supernatant of *P. aeruginosa* PAO1 was mixed with 0.3% azocasein, followed by sequential addition of 10% trichloroacetic acid and 1 M NaOH. The absorbance at 440 nm was measured to assess the protease activity [23].

Elastase was evaluated by combined the sterile supernatant with elastin Congo red buffer at 37 °C for 3 h. The reaction mixture was then centrifuged, and the resulting supernatant was collected for elastase determination by measuring its absorbance at 495 nm [24].

For pyocyanin quantification, *P. aeruginosa* PAO1 was grown in *Pseudomonas* broth overnight as described previously [13]. The cells were removed by centrifugation, and the OD of the resultant supernatant was measured at 695 nm.

Rhamnolipid was quantified by extracting the supernatant with two volumes of diethyl ether. The organic phase was collected and evaporated at 35 °C. The residue was dissolved in 100 μL of deionized water, followed by the addition of 900 μL of orcinol solution. The mixture was incubated at 80 °C for 30 min, and rhamnolipid was measured by reading OD_421_ [25].

### 2.3. Susceptibility and Biofilm Inhibition Assays

For susceptibility assay, *P. aeruginosa* PAO1 was inoculated into LB medium containing increasing concentration of Pip and EDTA. The mixtures were cultivated at 37 °C at 180 rpm for 24 h and the synergistic antibacterial activity was assessed by measuring OD_600_ [26].

For biofilm inhibition assay, *P. aeruginosa* PAO1 was grown in Tryptic Soy Broth (TSB) medium containing Pip and EDTA. After static culture overnight, the culture medium was discarded, and the formed biofilms were then sequentially processed with methanol fixation, crystal violet staining, and alcohol decolorization. Biofilms were quantified by reading OD_570_ [27].

To more directly visualize the inhibitory effect of the combination of Pip and EDTA on biofilm formation, SEM and CLSM were used. Briefly, biofilms of *P. aeruginosa* PAO1 were cultured on circular coverslips in 24-well plates. After cultivation, biofilms were sequentially processed through water washing, ethanol gradient dehydration, and freeze-drying. The lyophilized samples were sputter-coated with gold and then observed under SEM (Zeiss GeminiSEM 360, Carl Zeiss Microscopy GmbH, Jena, Germany) [28]. For CLSM analysis, biofilms formed on glass slides were first washed with phosphate-buffered saline (PBS) to eliminate planktonic cells. The attached biofilms were then subjected to staining with acridine orange (AO) and ethidium bromide (EB) and observed by CLSM (Zeiss LSM 700, Carl Zeiss Microscopy GmbH, Jena, Germany) [29].

### 2.4. Pull-Down Assay

The protein supernatant was collected by centrifugation following lysis of *P. aeruginosa* PAO1 cells. The protein samples were treated with biotin and Pip-biotin, respectively, followed by enzymatic digestion to generate peptides. The vacuum-dried peptides were reconstituted in 0.1% formic acid aqueous solution and subsequently separated via nanoflow liquid chromatography on a Thermo Scientific EASY-nLC 1000 system (Thermo Fisher Scientific, Waltham, MA, USA). The mass spectrometer (MS) model and detailed detection conditions were adopted from the experimental protocol of Hu et al. [30].

### 2.5. Transcriptional Analysis

RNA extraction was carried out utilizing the TRIzol^®^ method. RNA samples that passed quality inspection were sequenced using an Illumina HiSeq × TEN instrument (Illumina, Inc., San Diego, CA, USA). Read alignment was performed to the *P. aeruginosa* PAO1 complete genome sequence, followed by analysis of differentially expressed genes [21].

### 2.6. C4-HSL Production Analysis

Bacterial cells were grown in LB medium with or without Pip. After cultivation, the spent culture medium was centrifuged to obtain a sterile supernatant, which was then extracted using ethyl acetate. The solvent was removed in vacuo, and the residue was redissolved in methanol. The production of C4-HSL was quantified using liquid chromatography-tandem mass spectrometry (LC-MS/MS) [31].

### 2.7. Validation of Gene Expression

RNA extraction was carried out following the protocol for transcriptomics. RT-qPCR was performed to assess the transcriptional levels of key genes regulating QS and virulence (Table S1). The *gyrB* gene was used as an endogenous reference for data normalization in gene expression analysis [32].

### 2.8. Docking Analysis

The protein structure of RhlI was analyzed using the currently accepted highest-accuracy prediction model, AlphaFold2. The predicted structure is available at: https://www.uniprot.org/uniprotkb/P54291/entry#structure, accessed on 28 September 2024. (UniProt accession: P54291). Receptor and ligand preparation was performed using Discovery Studio software 4.0. The resulting docking scores were adopted to evaluate the binding affinity between Pip and its receptors RhlI [33].

### 2.9. Purification of RhlI

BL21 (DE3) cells were transformed with PET28a-*rhlI* and grown with 50 μg/mL of kanamycin to reach an OD value of 0.3. Protein expression was then induced by adding with 1 mM IPTG. The culture was subsequently grown overnight at 16 °C with continuous shaking. Bacterial cells were pelleted and resuspended in 50 mM PBS containing 0.1 mM phenylmethanesulfonyl fluoride (PMSF) and then subjected to ultrasonication at 4 °C. After centrifugation, the supernatant was collected and mixed with Ni-NTA agarose beads for 1 h. Proteins were eluted with PBS containing 300 mM imidazole. The presence of target proteins in harvested solutions was validated by SDS-PAGE with protein molecular weight ladders (Blue Plus Protein Marker, TransGen Biotech, Beijing, China) as references, followed by microvolume spectrophotometry for protein concentration detection.

### 2.10. Binding Affinity Assay

Isothermal titration calorimetry (ITC) assay was conducted on a PEAQ-ITC instrument at 25 °C using a 50 mM PBS (pH 7.2). The initial doses of proteins and Pip were set at 26 μM and 2 mM, respectively.

### 2.11. Gene and Amino Acid Site Mutation

Gene knockout was performed employing the homologous recombination method, with the plasmid pK18 as the vector. PCR amplification of the target gene flanking regions was carried out using *P. aeruginosa* PAO1 genomic DNA as the template. The product was purified and digested with enzyme. The gentamicin resistance marker along with its promoter was amplified by PCR from the plasmid pBBR1MCS-5. The resulting PCR product was purified and digested with restriction endonucleases. The PCR product and the pK18 vector were digested with restriction enzymes and ligated using T4 DNA ligase. The ligated DNA was transformed into *Escherichia coli* competent cells. Subsequently, the transformation mixture was plated on tetracycline-supplemented LB agar and incubated overnight at 37 °C, allowing for selection of recombinant clones. The plasmid was extracted and verified by sequencing [34].

For amino acid site mutation assay, cDNA primer sequences were designed based on the amino acid binding sites predicted by molecular docking. The detailed experimental steps for amino acid site-directed mutagenesis were performed as described for gene mutagenesis. A summary of the plasmids utilized is provided in Table S2.

### 2.12. Oxidative Stress and Membrane Permeability

For reactive oxygen species (ROS) determination, the harvested cells were incubated with 6-carboxy-2′,7′-dichlorodihydrofluorescein diacetate for 30 min. After incubation, cells were collected and resuspended in PBS. ROS was determined using a fluorescence spectrophotometer (Hitachi, Tokyo, Japan) [35].

The outer membrane permeability was determined by adding 500 μM N-Phenyl-1-naphthylamine (NPN) to the Pip- and EDTA-treated samples. Following a 30-min incubation in the dark, the fluorescence intensity of each treatment group was determined using a fluorescence spectrophotometer (λex = 350 nm, λem = 420 nm) [36].

The inner membrane permeability was determined by adding 1 mM propidium iodide (PI) to the treated samples. After incubation in the dark for 30 min, an aliquot (1 mL) of the incubated culture was taken, and the fluorescence intensity was measured (λex = 535 nm, λem = 617 nm) [36].

For protein leakage analysis, *P. aeruginosa* PAO1 was treated with Pip, EDTA, or their combination overnight. Following incubation, the supernatants were collected by centrifugation, and protein concentrations were measured using a Bradford Protein Assay Kit as described previously [37].

For nucleic acid leakage analysis, culture supernatants from different treatment groups were collected by centrifugation, filtered through a 0.22 μm membrane, and then analyzed for OD_260_ using a UV spectrophotometer [37].

### 2.13. Toxicological and Anti-Infection Evaluation

The toxicological effect was evaluated by inoculating 20 adult *Caenorhabditis elegans* into LB medium with or without Pip. Following a 24-h incubation period, the number of viable nematodes was quantified to assess the potential toxicity of Pip.

In parallel, the anti-infection activity of Pip against *P. aeruginosa* PAO1 was evaluated using the *C. elegans* infection model as described previously [38]. Briefly, adult nematodes (20 per group) were co-cultured with *P. aeruginosa* PAO1 at 28 °C for 12 h. After cultivation, the infected nematodes were washed with M9 buffer and subsequently transferred to LB medium supplemented with or without Pip, followed by incubation at 28 °C for 32 h. The number of viable nematodes in each treatment group was determined.

### 2.14. Statistical Analysis

All experiments were performed with replicates. Statistical analyses were conducted using SPSS software (version 18.0), and a *p*-value of less than 0.05 was considered statistically significant.

## 3. Results

### 3.1. MIC and Growth Profile

**Figure 1:**
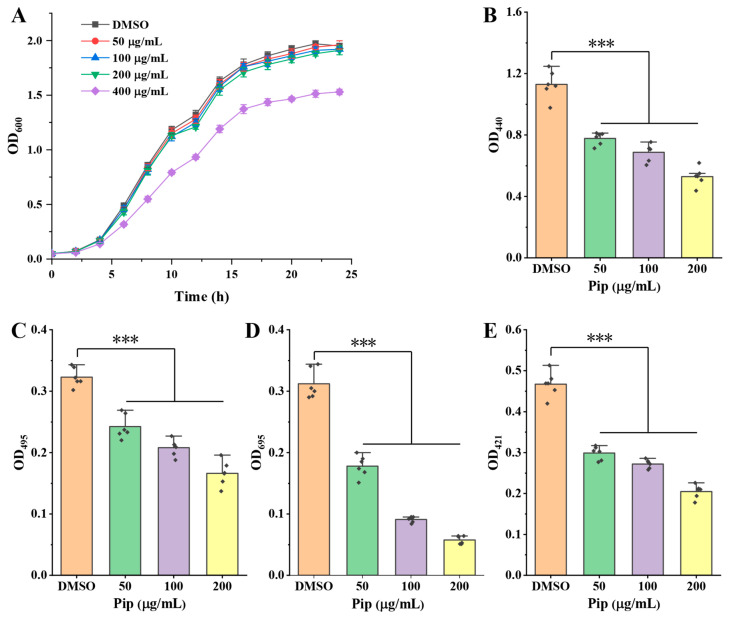
Anti-virulence activity of Pip against *P. aeruginosa* PAO1. (**A**) Growth profile of *P. aeruginosa* PAO1 following exposure to Pip. (**B**–**E**) Inhibitory effect of Pip on virulence factors of protease, elastase, pyocyanin, and rhamnolipid, respectively. (∗∗∗) *p* < 0.001.

The MIC of EDTA and Pip against *P. aeruginosa* PAO1 was determined to be 2 mg/mL and greater than 2 mg/mL, respectively. Growth analysis showed that Pip exposure at 50, 100 and 200 μg/mL had no inhibitory effect on cells growth (Figure 1A). However, exposure to Pip at 400 μg/mL exerts marked growth inhibition (Figure 1A). Since a bona fide QSI should not impede bacterial growth [39], sub-inhibitory concentrations of 50, 100, and 200 μg/mL were selected to assess its anti-virulence activity.

### 3.2. Effect of Pip on Virulence Factors Production

As shown in Figure 1B,C, the activities of protease and elastase were notably decreased following Pip exposure. The residual activity of protease was about 47% following treatment with 200 μg/mL of Pip. Pip treatment at doses of 50 to 200 μg/mL caused 25%, 36%, and 49% reduction in elastase, respectively, compared with the DMSO-treated group. Pip was also found to inhibit the secretion of pyocyanin in a dose-dependent fashion (Figure 1D). Strikingly, treatment with Pip resulted in a 43%, 71%, and 81% reduction in pyocyanin production at 50, 100, and 200 μg/mL, respectively. The effect of Pip on rhamnolipid production was presented in Figure 1E. After Pip treatment, rhamnolipid levels were reduced by 36%, 42%, and 56%, respectively.

### 3.3. Synergistic Inhibition of Growth and Biofilm Formation by Pip and EDTA

**Figure 2:**
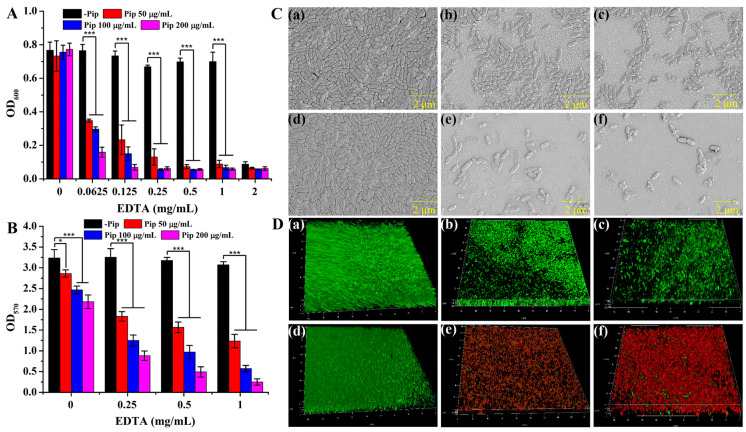
Effects of Pip on EDTA susceptibility and biofilm inhibition. (**A**,**B**) represent synergistic effect of Pip and EDTA on bacterial growth and biofilm formation of *P. aeruginosa* PAO1, respectively. (**C**,**D**) represent SEM and CLSM images of biofilms treated with (**a**) DMSO, (**b**) 100 μg/mL of Pip, (**c**) 200 μg/mL of Pip, (**d**) 1 mg/mL of EDTA, (**e**) 100 μg/mL of Pip + 1 mg/mL of EDTA, and (**f**) 200 μg/mL of Pip + 1 mg/mL of EDTA, respectively. (∗) *p* < 0.05 and (∗∗∗) *p* < 0.001.

As shown in Figure 2A, the MIC of EDTA against *P. aeruginosa* PAO1 was 2 mg/mL. However, its antibacterial activity was significantly potentiated by the addition of Pip. Specifically, the MIC of EDTA was reduced to 0.5, 0.25, and 0.125 mg/mL in the presence of 50, 100, and 200 μg/mL Pip, respectively. Furthermore, the reduction in OD values indicated that Pip, despite not reducing the MIC of EDTA any further, substantially potentiated its antibacterial effect at low concentrations.

The results of the biofilm inhibition assay showed that Pip exposure at 50, 100 and 200 μg/mL reduced biofilm formation by approximately 12%, 24% and 33%, respectively, whereas EDTA alone showed no significant inhibitory activity (Figure 2B). However, the inhibitory activity of EDTA was markedly potentiated in the presence of Pip. Biofilm formation was reduced by 43%, 61% and 73% when 0.25 mg/mL EDTA was combined with 50, 100 and 200 μg/mL of Pip, respectively. Elevating the EDTA concentration to 1 mg/mL resulted in a 92% inhibition of biofilm formation, indicating synergy with 200 μg/mL of Pip.

SEM analysis indicated that cells in the DMSO-treated group exhibited a highly dense, mesh-like structure embedded within abundant extracellular matrix (Figure 2C(a)). However, treatment with Pip caused a scattered appearance and disrupted integrity of the biofilms (Figure 2C(b,c)). Although EDTA monotherapy showed no significant reduction in biofilm formation quantitatively, the resulting biofilm exhibited a porous, loosened morphology with noticeably less surface colonization (Figure 2C(d)). When treatment with EDTA in combination with Pip, an extensive degradation of the biofilms was observed. The characteristic dense, mushroom-shaped structures were absent, replaced by a sparse layer of cells with compromised membranes and negligible extracellular matrix (Figure 2C(e,f)), underscoring the potent synergistic anti-biofilm activity of the compound combination.

In addition, a similar biofilm-diminishing phenotype was exhibited upon co-administration of EDTA and Pip, as visualized by CLSM. Upon Pip treatment, the biofilm thickness decreased from 13.1 ± 0.46 μm (Figure 2D(a)) to 5.14 ± 1.02 μm (Figure 2D(c)). Furthermore, the EDTA-Pip combination correlated with direct antimicrobial penetration and consequent massive cell death, as conclusively evidenced by the extensive red fluorescence corresponding to dead cells (Figure 2D(e,f)).

### 3.4. Identification of RhlI as Potential Target of Pip

**Figure 3:**
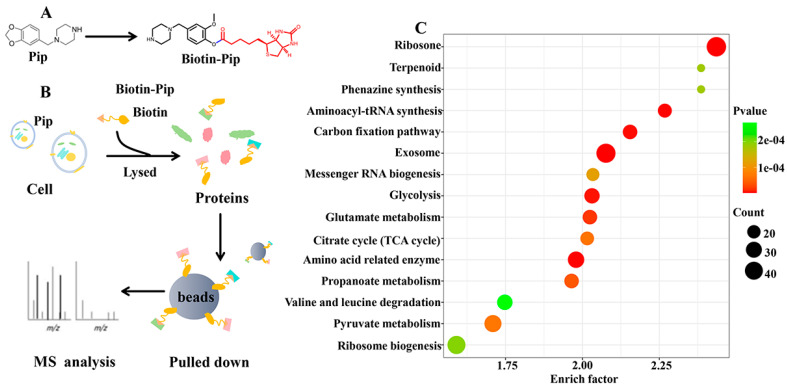
Utilization of the pull-down approach to identify the potential target protein of Pip. (**A**) Molecular structures of Pip and biotin-labelled Pip (Biotin-Pip). (**B**) Schematic representation of the detection of Pip-binding proteins using a pull-down assay. (**C**) KEGG pathway of Pip-binding proteins.

To elucidate the mechanism by which Pip reduces virulence and enhances efficacy, a biotin-pull-down MS strategy was employed (Figure 3B). Pip-Biotin was chemically synthesized (Figure 3A) and characterized by NMR and MS spectrometric analyses (Figure S1). A total of 1663 proteins that potentially interact with Pip-Biotin were assigned using MS analysis following the pull-down assay. The top 15 enriched KEGG pathways were primarily associated with phenazine synthesis, glutamate metabolism and energy metabolism (Figure 3C). Results from both pull-down assay and virulence-attenuating synergy experiments suggested that Pip may bind to RhlI (Coverage rate = 50%, Unipeptide = 27) (Table S3) as this protein is critically involved in virulence factors production in *P. aeruginosa* [40,41].

**Figure 4:**
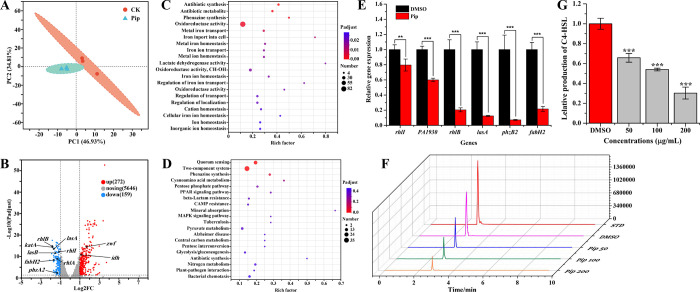
Effect of Pip on gene expression and C4-HSL production. (**A**–**D**) depict the PCA plot, volcano plot, GO annotation, and KEGG enrichment analysis plot, respectively, based on the transcriptomic data. (**E**) Validation of gene expressions using RT-qPCR. (**F**,**G**) represent quantification of C4-HSL production treated with Pip. (∗∗) *p* < 0.01 and (∗∗∗) *p* < 0.001.

To further elucidate the mechanism of action and uncover potential cellular targets of Pip, transcriptome profiling was conducted. The result indicated that Pip treatment significantly reprogrammed the gene expression profile of *P. aeruginosa* (Figure 4A), leading to upregulation of 272 genes and downregulation of 159 genes (Figure 4B). GO enrichment analysis indicated that Pip exposure lead to distinguished impact on antibiotic metabolism, virulence factors synthesis and membrane transport (Figure 4C). To functionally annotate the differentially expressed genes (DEGs), we also conducted a KEGG analysis to delineate their associated biological processes and pathways. A total of 431 DEGs were successfully mapped to corresponding KEGG pathways, and they were significantly enriched in 156 distinct categories. The top 20 significantly enriched KEGG pathways were screened to identify crucial candidate genes, with several DEGs proved to be involved in QS, virulence factor synthesis, and drug resistance (Figure 4D). The detailed DEGs and their corresponding changes in expression level were present in Table S4.

As presented in Table S4, Pip treatment markedly reduced the transcription of virulence genes *lasA*, *lasB*, *rhlA*, *rhlB* and *phzB2*, which encode protease, elastase, rhamnolipids and pyocyanin, respectively. This transcriptional repression was consistent with the observed reduction in actual virulence factor production, thereby confirming the anti-virulence activity of Pip. Given that these genes are under QS control, their suppressed expression suggested a potential disruption of the QS system [42]. This hypothesis was corroborated by the specific downregulation of *rhlI*, indicating that Pip likely exerts its anti-virulence effects by targeting *rhlI*.

### 3.5. Gene Expression Validation and C4-HSL Production Analysis

To validate the transcriptomic data, 6 DEGs enriched in specific KEGG pathways were subjected to relative expression quantification through RT-qPCR. The result indicated that the expressions of *rhlI*, *PA1930*, *rhlB*, *lasA*, *phzB2*, and *fabH2* were significantly down-regulated after Pip treatment (Figure 4E). A high degree of concordance was observed between the RT-qPCR results and the RNA-seq data, thus confirming the reproducibility of the transcriptome sequencing.

Additionally, as the synthesis of the C4-HSL autoinducer is directly regulated by *rhlI* [5], we posited that the transcriptional inhibition of *rhlI* would lead to a deficit in the corresponding signaling molecule. To test this premise, the concentration of C4-HSL secreted by *P. aeruginosa* was quantified. The data revealed a significant attenuation of C4-HSL synthesis upon Pip exposure (Figure 4F,G), thereby mechanistically corroborating the model that Pip functions as a specific inhibitor of the *rhlI* gene.

### 3.6. Pip Inhibiting RhlI by Binding Directly to Its LYS-164 and ASP-35 Sites

**Figure 5:**
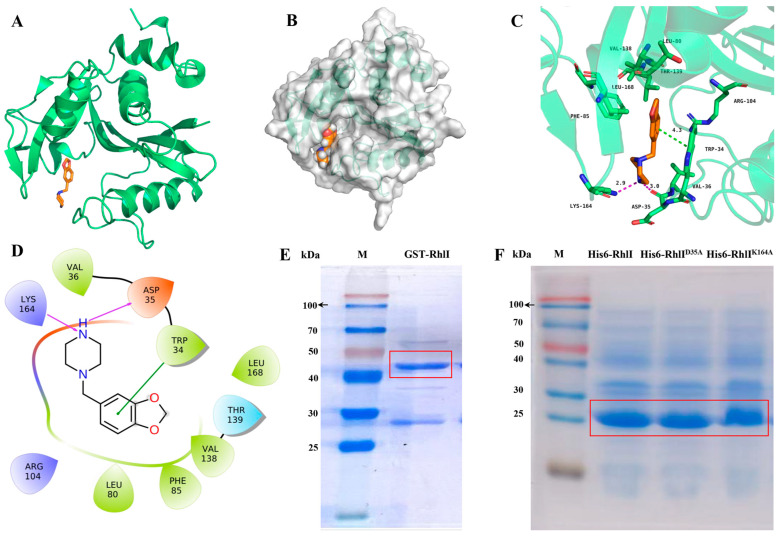
The binding interaction between Pip and RhlI by molecular docking and the purification of RhlI-related proteins. (**A**) Binding mode of Pip with RhlI was present in cartoon representation. (**B**) Binding mode of Pip with RhlI was present in surface representation. (**C**,**D**) represent 3D and 2D schematics of receptor-ligand interactions of RhlI with Pip using molecular docking. (**E**,**F**) represent purification of RhlI-related proteins.

**Figure 6:**
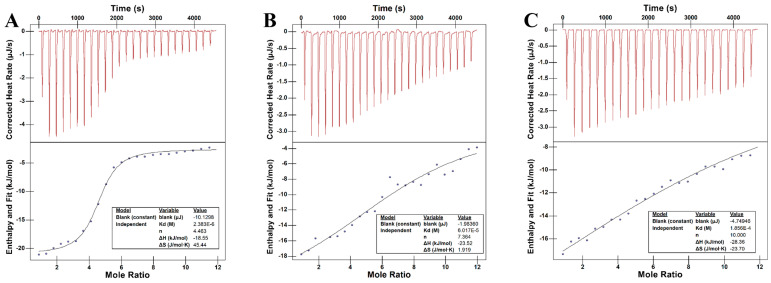
Affinity studies between Pip and RhlI by ITC. (**A**) represents kinetic binding profiles of Pip with RhlI. (**B**,**C**) represent binding affinity between Pip and RhlI with LYS-164 and ASP-35 mutations, respectively.

In silico docking simulations were carried out to forecast the potential interactions, including binding affinity and binding sites, between Pip and RhlI. As shown in Figure 5A,B, the small molecule Pip is docked into the middle of a binding groove on RhlI. Further analysis of the binding details revealed that Pip engages in hydrogen bonding interactions with Lys164 and Asp35 of RhlI (Figure 5C,D), with a binding energy of −6.13 kcal/mol and a predicted dissociation constant (Kd) of 31.91 μM. To further confirm the binding interaction between Pip and RhlI, we purified both wild-type RhlI (Figure 5E) and its mutant variants generated by site-directed mutagenesis (Figure 5F). Subsequently, the binding affinity was quantified by ITC, with the Biacore software (V3.0) analysis returning a Kd of 2.38 μM (Figure 6A). Collectively, these findings reveal that Pip possesses strong binding affinity toward the RhlI protein.

To experimentally validate the predicted binding residues LYS-164 and ASP-35 from the docking analysis, we generated alanine substitutions. The binding affinity of Pip for the mutant proteins, as quantified by ITC, was markedly reduced, with Kd values of 185.60 (Figure 6B) and 60.17 μM (Figure 6C), respectively. This strongly suggests that Pip is a novel RhlI inhibitor targeting LYS-164 and ASP-35.

**Figure 7:**
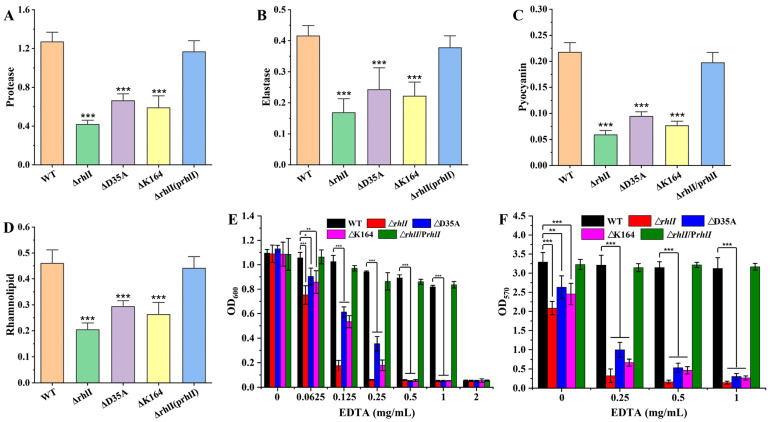
Effect of *rhlI* deletion and LYS-164/ASP-35 mutations on the production of protease (**A**), elastase (**B**), pyocyanin (**C**), and rhamnolipid (**D**) and their effect on EDTA susceptibility (**E**) and biofilm inhibition (**F**). (∗) *p* < 0.05, (∗∗) *p* < 0.01, and (∗∗∗) *p* < 0.001.

The anti-virulence activity assessment revealed that the deletion of the *rhlI* gene significantly reduced the production of protease, elastase, pyocyanin, and rhamnolipid in *P. aeruginosa* (Figure 7A–D). However, the production of these virulence factors was largely restored to the wild-type level upon genetic complementation (Figure 7A–D). In addition, consistent with the loss of binding, mutation of LYS-164 and ASP-35 in RhlI also impaired the biosynthesis of these virulence determinants (Figure 7A–D).

Antimicrobial susceptibility testing showed that upon deletion of the *rhlI* gene, the MIC of EDTA against *P. aeruginosa* decreased from 2 mg/mL to 0.25 mg/mL (Figure 7E), representing a 7-fold increase in susceptibility to EDTA. Meanwhile, mutations at the RhlI amino acid residues LYS-164 and ASP-35 also significantly enhanced EDTA susceptibility, with a 3-fold increase for both mutants (Figure 7E). Complementation of the deleted *rhlI* gene largely restored the EDTA MIC to the level of the wild-type strain (Figure 7E). Biofilm inhibition assays yielded results consistent with susceptibility testing: deletion of *rhlI* or mutation of LYS-164 and ASP-35 markedly inhibited biofilm formation in *P. aeruginosa* and potentiated the antibiofilm activity of EDTA (Figure 7F).

### 3.7. Oxidative Stress and Membrane Integrity

**Figure 8:**
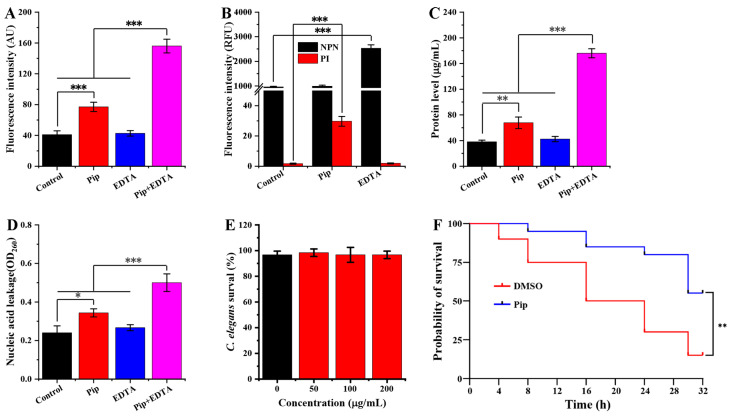
Evaluation of Pip in relation to oxidative stress (**A**), membrane permeability (**B**), protein leakage (**C**), nucleic acid leakage (**D**), toxicity against *C. elegans* (**E**), and anti-infective efficacy against *C. elegans* (**F**). (∗) *p* < 0.05, (∗∗) *p* < 0.01, and (∗∗∗) *p* < 0.001.

To determine whether Pip contributes to oxidative damage, ROS production was assessed. The results showed that Pip treatment significantly upregulated ROS synthesis (Figure 8A), confirming its role in inducing oxidative stress. Additionally, EDTA exhibited no intrinsic capacity to induce ROS (Figure 8A); however, co-administration with Pip led to a dramatic surge in ROS levels (Figure 8A), demonstrating a synergistic enhancement of the cellular stress response.

The permeability assay demonstrated that EDTA significantly increased NPN fluorescence, while Pip markedly enhanced PI fluorescence (Figure 8B). Since NPN and PI are indicators of outer and inner membrane integrity [36], respectively, these results point to an EDTA-induced increase in outer membrane permeability and a Pip-induced increase in inner membrane permeability.

To further evaluate membrane integrity and permeability, we measured protein and nucleic acid leakage following Pip and EDTA treatment. Results showed that Pip significantly increased leakage of both proteins (Figure 8C) and nucleic acids (Figure 8D), whereas EDTA alone had no notable effect. Strikingly, the combination of Pip and EDTA caused a substantial increase in leakage. These results indicate that Pip disrupts membrane integrity and permeability, and that its combination with EDTA produces a synergistic effect.

### 3.8. Toxicological and Anti-Infection Evaluation

Toxicological assays indicated that Pip exhibited no nematocidal activity against *C. elegans* at concentrations of 50–200 μg/mL (Figure 8E). *C. elegans* has been extensively validated as a predictive model for mammalian toxicity and environmental biosafety assessment [43]. The absence of lethal effects suggests that Pip exhibits a favorable safety profile in this model system at the concentrations tested. To investigate Pip’s virulence-restricting activity, we utilized the *C. elegans* fast-killing infection model. It was observed that nematode mortality reached 85% following a 30-h incubation period (Figure 8F), thereby establishing the considerable virulence of *P. aeruginosa* in this model. In contrast, much less killing occurred when the nematodes were exposed to Pip (Figure 8F), indicating its strong anti-infection potential.

## 4. Discussion

EDTA is an important, safe, and cost-effective aminopolycarboxylic acid that exhibits antimicrobial activity against a range of foodborne pathogens and is widely employed as a food additive [17]. Despite these advantages, the antimicrobial efficacy of EDTA alone is relatively weak. It functions primarily as an antimicrobial potentiator by chelating divalent cations and destabilizing the outer membrane of Gram-negative bacteria [44]. This limitation restricts its standalone application in food preservation, creating an urgent need for strategies to enhance its antimicrobial activity. Piperonylpiperazine (Pip), a principal bioactive component of pepper with established antitumor and neuroprotective properties [19,45], represents a promising candidate for investigation. However, its anti-virulence activity against foodborne *P. aeruginosa* and its potential to potentiate EDTA have not yet been explored. Thus, the present study aims to systematically evaluate the anti-virulence and EDTA-potentiating activities of Pip, assess its efficacy in preserving chicken meat, and elucidate the mechanisms by which it attenuates virulence and enhances EDTA activity.

*P. aeruginosa* produces a battery of virulence factors including protease, elastase, pyocyanin, and rhamnolipid. Protease, whose synthetic machinery is encoded by the *lasA* gene, accumulates at high transcriptional levels to foster the development of robust, fully mature biofilm structures. These multilayered biofilms create a physical shield that restricts the permeation of antimicrobials, drastically elevating the drug resistance phenotype of bacteria trapped within the matrix [46]. Elastase, the protein product of *lasB*, exerts indirect control over biofilm development by modulating the synthetic output of rhamnolipids, which in turn augments the pathogen’s intrinsic tolerance to various antibiotics [47]. Rhamnolipids, biosynthesized through the enzymatic products of *rhlA* and *rhlB*, possess potent surface-active characteristics that stabilize interconnected aqueous channels across biofilm interiors. These channels permit efficient exchange of nutrients and gaseous substances while initiating programmed cell death, jointly establishing both physical and physiological defensive barriers that hinder polymyxin penetration and reinforce bacterial polymyxin tolerance [10,48]. Pyocyanin, one of the most prominent virulence pigments released by *P. aeruginosa*, depends on the regulatory activity of *phzB2* for its biogenesis. This metabolite stimulates extracellular DNA liberation to consolidate biofilm integrity and further intensify the strain’s antibiotic-resistant phenotype [10]. Intriguingly, the production of all the above virulence effectors falls under the global regulatory governance of the QS circuitry. This QS network not only orchestrates the expression of these pathogenic determinants but also modulates bacterial invasion, antibiotic resistance, pathogenicity, and spoilage activity in meat products [21]. Therefore, the suppression of these virulence factors serves as a hallmark of QS interference. In this study, the significant decrease in the production of virulence factors suggested that Pip likely interferes with the QS system of *P. aeruginosa*. To test this hypothesis and uncover its molecular target, we conducted a combination of pull-down and transcriptomic profiling. These approaches revealed that Pip directly targets RhlI, a crucial synthase in the QS pathway, by both binding to the protein and repressing its gene expression. The consequent sharp decline in C4-HSL levels further corroborated RhlI as the primary target. To precisely map the interaction, we employed molecular docking followed by rigorous biophysical validation using MST, SPR, and ITC on both wild-type and mutant RhlI proteins. Our results demonstrate that Pip binds RhlI with high affinity at specific amino acid residues, LYS-164 and ASP-35, leading to the inhibition of its activity. The physiological relevance of this interaction was confirmed by gene knockout experiments, wherein deletion of *rhlI* or mutation of these critical residues phenocopied the effects of Pip treatment, resulting in markedly reduced virulence factor synthesis. Taken together, these findings establish that Pip exerts its anti-virulence effect by specifically targeting RhlI, offering a promising strategy for combating *P. aeruginosa* pathogenicity and spoilage activity.

Biofilms serve as a major determinant of *P. aeruginosa* tolerance to antimicrobial preservatives [49]. The biofilm extracellular polymeric substances construct a dense, protective fortress that envelops bacterial communities [21]. This architectural matrix functions as a physical diffusion barrier, effectively restricting antimicrobial penetration and thereby enabling bacterial survival under otherwise lethal conditions [21]. Thus, targeting biofilm formation has emerged as a pivotal approach to combat *P. aeruginosa* resistance by dismantling this protective shield. Extensive evidence indicates that biofilm formation in *P. aeruginosa* is governed by QS, which orchestrates the expression of matrix components and coordinates biofilm maturation [5,50]. Strains with defective QS systems produce substantially less biofilm biomass, and the residual biofilms exhibit a loose, disorganized architecture with a tendency to detach from surfaces [51]. Based on our finding that Pip effectively inhibits the QS system of *P. aeruginosa*, we postulated that Pip might also impair biofilm development. Subsequent crystal violet quantification assays revealed a marked reduction in biofilm formation following Pip treatment. SEM observation further corroborated these findings, demonstrating that Pip-treated biofilms were flat and loose in structure, with sparse extracellular polymeric substances, closely resembling the phenotype of QS-deficient mutants.

The intrinsic tolerance of *P. aeruginosa* biofilms to conventional antimicrobials and preservatives poses a significant challenge to food safety and human health [4]. Given the well-documented role of the biofilm phenotype in this recalcitrance and the previously demonstrated antibiofilm activity of Pip, we investigated its potential as an adjutant to enhance preservative efficacy. Our initial hypothesis—that Pip would sensitize *P. aeruginosa* to conventional agents—was confirmed through a pronounced synergistic interaction with EDTA. While neither compound alone affected planktonic growth at sub-inhibitory concentrations, their combination resulted in a striking 16-fold reduction in the MIC of EDTA. This potentiation extended to pre-formed biofilms, where Pip significantly bolstered EDTA’s eradicating activity, highlighting a combinatorial effect that targets both planktonic and sessile populations. Mechanistically, this synergy appears to be orchestrated through a multi-pronged attack on bacterial integrity. We posit that Pip facilitates the disruption of the biofilm matrix, creating a conducive environment for EDTA to exert its known permeabilizing effects on the outer membrane [18]. This combined assault likely induces substantial oxidative damage and compromises membrane integrity, leading to the observed cell death. Central to this model is the role of rhamnolipids, biosurfactants critical for maintaining the fluid channels and three-dimensional architecture of mature *P. aeruginosa* biofilms [52]. Our data suggest that Pip disrupted rhamnolipid homeostasis. The consequent reduction in this key structural component inevitably leads to biofilm collapse. This was corroborated by quantitative biomass measurements and SEM, which revealed a transition from a robust, structured biofilm to a loose, disorganized cellular aggregate following treatment. These findings position the Pip-EDTA combination as a promising dual-action strategy that both directly kills bacteria and dismantles their protective fortress.

**Figure 9:**
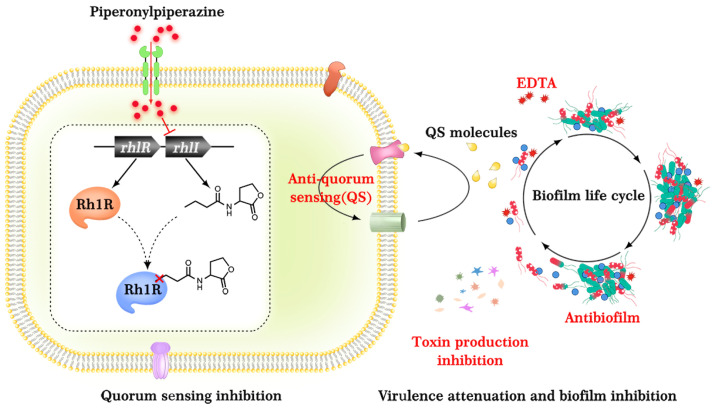
Mechanism of Pip-mediated virulence attenuation and EDTA potentiation in *P. aeruginosa*.

The intricate interplay between QS and oxidative stress responses is increasingly recognized as a critical determinant of bacterial survival under hostile conditions [53]. Prior work has established that the *rhl* QS system, particularly via C4-homoserine lactone signaling, governs the expression of antioxidant enzymes such as catalase, with *rhlI* mutants displaying heightened sensitivity to oxidative insults [54]. Our finding that Pip represses *rhlI* expression prompted us to investigate its pro-oxidant potential. Indeed, Pip treatment elicited a marked elevation in intracellular ROS, implicating oxidative stress as a component of its mechanism. Notably, EDTA, while inert alone, synergistically amplified Pip-induced ROS accumulation, suggesting that the combination overwhelms bacterial antioxidant capacity. Oxidative stress is well-documented to perturb membrane homeostasis, altering lipid architecture and permeability, thereby facilitating antimicrobial entry [21]. Consistent with this paradigm, we observed that EDTA selectively compromised outer membrane integrity, whereas Pip disrupted the inner membrane. This compartmentalized attack resulted in substantial leakage of proteins and nucleic acids, effects that were profoundly enhanced by combination therapy. Structural correlates of this biochemical disruption were visualized by CLSM, wherein the surge in PI-red cells signified extensive loss of membrane integrity [55]. Synthesizing these observations, we propose a hierarchical mechanism wherein Pip-mediated quorum quenching of the *rhl* system initiates a cascade: impaired antioxidant defense → ROS accumulation → oxidative membrane damage → enhanced permeability → increased antimicrobial susceptibility (Figure 9). This model is congruent with the observed synergy between Pip and EDTA against both planktonic and biofilm populations, and aligns with emerging paradigms that position QS inhibitors as adjuvants capable of re-sensitizing resistant pathogens.

A prerequisite for any compound intended as a food preservative is demonstrable safety for human consumption. The fact that Pip is a naturally occurring constituent of the dietary spice pepper substantiates its safety for consumption. To rigorously test this premise, we leveraged the nematode *C. elegans* as a predictive in vivo model for mammalian safety assessment. Our result revealed that Pip, across a concentration gradient of 50–200 μg/mL, elicited no detectable increase in nematode mortality relative to untreated controls, establishing a no-observed-adverse-effect level within this range and substantiating its safety profile for potential food applications. Importantly, the utility of *C. elegans* extends beyond safety pharmacology to encompass infectious disease modeling, where it serves as a surrogate host for studying pathogen virulence in vivo [56]. Our result indicated that Pip treatment conferred significant protection against *P. aeruginosa*-mediated killing, as evidenced by markedly reduced nematode mortality. These findings corroborate the work of Xu et al. [38] and reinforce the emerging paradigm that QSIs represent a promising anti-virulence strategy for disarming pathogens without exerting overt selective pressure.

The anti-virulence effect of Pip characterized herein relies on its inhibitory activity toward the RhlI synthase belonging to the LuxI/LuxR-type QS circuit, a regulatory module widely conserved across diverse Gram-negative pathogens. Homologous autoinducer synthases exist in numerous foodborne and clinical pathogens, including *Serratia marcescens*, *Chromobacterium violaceum*, and *Burkholderia cepacia*, implying that Pip may exert broad-spectrum anti-QS activity beyond *P. aeruginosa*. Structural similarities in the ligand-binding pockets of LuxI-type proteins make it plausible that Pip could interfere with signal molecule synthesis in these species, yet interspecific differences in enzyme amino acid sequences and QS regulatory networks may create distinct susceptibility thresholds, restricting universal efficacy.

Several limitations should be acknowledged to contextualize the present results. All phenotypic and transcriptional assays were performed exclusively with the laboratory reference strain *P. aeruginosa* PAO1; clinical isolates with varied genetic mutations and drug-resistant backgrounds were not tested, nor were other Gram-negative pathogens included for mechanistic verification. In addition, the anti-virulence and synergistic effects of Pip combined with EDTA have only been validated in in vitro culture systems, without further assessment in mammalian infection models to confirm in vivo therapeutic feasibility. Future work will systematically screen representative Gram-negative pathogens and clinical *P. aeruginosa* isolates, and construct animal infection models to clarify the cross-species anti-QS spectrum and in vivo biocontrol potential of Pip.

## 5. Conclusions

This study evaluated the anti-virulence and EDTA-sensitizing effects of Pip from pepper against *P. aeruginosa* and its application in anti-infective therapy against *C. elegans*. Mechanistically, Pip targets the QS circuitry by binding to the Lys-164 and Asp-135 residues of the RhlI synthase, thereby disrupting its catalytic function. This disruption exacerbates oxidative cellular damage, compromising the cell membrane structure and increasing its permeability. The resulting enhanced permeability facilitates greater EDTA efficacy, ultimately attenuating the pathogenicity of *P. aeruginosa* towards *C. elegans*. Given its safety in *C. elegans* and its preservative effects, Pip is a promising novel agent to control foodborne *P. aeruginosa*.

## Data Availability

The original contributions presented in this study are included in the article and Supplementary Materials. Further inquiries can be directed to the corresponding authors.

## Supplementary Materials

The following supporting information can be downloaded at: https://www.mdpi.com/article/10.3390/foods15152660/s1, Table S1. Primers Used in This Study; Table S2. Strains and Plasmids in This Study; Table S3. Candidate Proteins for Pip Binding were Identified by Comprehensive Analysis Using the Uniprot Database; Table S4. Selected DEGs related to virulence, energy metabolism and oxidative stress; Figure S1. MS (A) and NMR (B) Spectra of Pip-Biotin.
