# Supplementary material for: Piperonylpiperazine Targets RhlI to Reduce Virulence and Potentiate EDTA Sensitivity in Pseudomonas aeruginosa
Source: Foods. 2026 Jul 29;15(15):2660. doi: 10.3390/foods15152660 (PMC13465478; doi:10.3390/foods15152660)
Supplement: Supplementary file 1 [file foods-15-02660-s001.zip › foods-4438929-supplementary.pdf]

Article

Piperonylpiperazine Targets RhlI to Reduce Virulence and Po-tentiate EDTA Sensitivity in Pseudomonas aeruginosa

Jin-Wei Zhou a, *, Yu-Xin Qiu a, Hai-Yan Wang a, Meng Chen a, Jia-Wen Li a, Gu-Yitian Xu a, Jia-Cheng Liu a, Xin-Yu Wu a, Yao Zou a, Ying Wang a, Xiao-Juan Tan b, Xin-Yu Qian c, *, and Jin-Fang Zhou c

aSchool of Food and Biological Engineering, Xuzhou University of Technology, Xuzhou 221018, China

bAnhui Provincial Key Laboratory of Molecular Enzymology and Mechanism of Major Diseases, College of Life Sciences, Anhui Normal University, Wuhu 241000, China

cShaanxi Key Laboratory of Agricultural and Environmental Microbiology, College of Life Sciences, Northwest A&F University, Yangling 712100, China

* Correspondence: E-mail address: zhoujinwei@xzit.edu.cn(J.-W. Zhou), 18263579003@163.com(X.-Y. Qian)

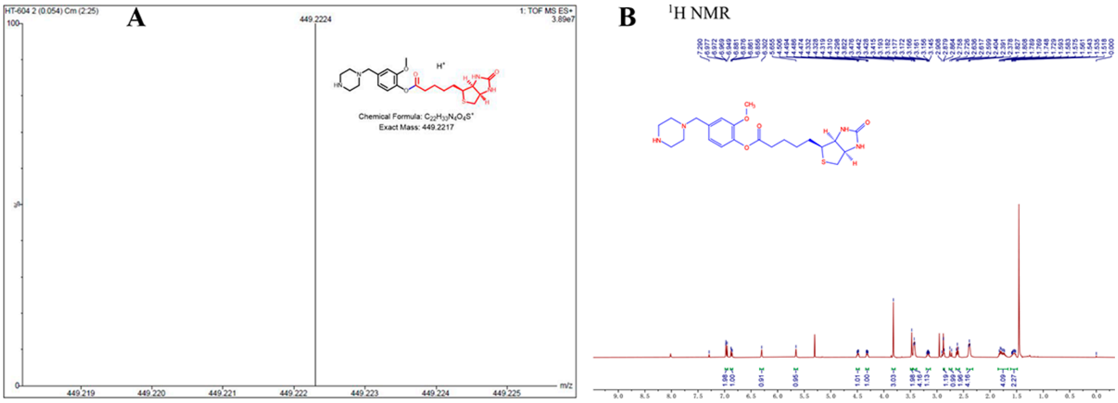

Figure S1. MS (A) and NMR (B) Spectra of Pip-Biotin.

Table S1. Primers Used in This Study.

Primer	5'-3' sequence	Purpose
pK18-F	TTCCGGCTCGTATGTTGTGTGG	To detect knockout plasmid
pK18-R	GCGTGAAGCTAGCTTATCGCC	
rhlI-F-BamHI	CGGGATCCATGATCGAATTGCTCTCTGAATCG	Forward primer to clone *rhlI* into pET28a
rhlI-R-HindIII	CCCAAGCTTTCACACCGCCATCGACAGC	
rhlI-UP-F-XbaI	GCTCTAGAATCCGATGCTGATGTCCAACCC	To amplify the downstream of *rhlI* from strain PAO1 for fusion
rhlI-UP-R	TACGCCCTGCAGCCATTCCGCGATCATGGCGGC	
rhlI-Down-F	CTTTCCGCCGCCATGATCGCGGAATGGCTGCAG	
rhlI-Down-R-HindIII	CCCAAGCTTGTCGCGCAGGTAGGGAATCG	
rhlI-UP-F-*BamHI*	CGGGATCCATGATCGAATTGCTCTCTGAATCGC	Forward primer to clone *rhlI*^{D35A} /*ΔrhlI*^{K164A} into pET28a/pGEX-6P-1
rhlI-D35A-UP-R	GGAGACCACGGCCCAGCC	
rhlI-D35A-Down-F	GGCTGGGCCGTGGTCTCC	
rhlI-K164A-UP-R	GTCTCGCCCGCGACCTTCT	
rhlI-K164A-Down-F	AGAAGGTCGCGGGCGAGAC	
rhlI-Down-R-XhoI	CCGCTCGAGTCACACCGCCATCGACAGC	
pBBR1MCS-5-F	TTAGGCACCCCAGGCTTTACA	To detect mutant strains
pBBR1MCS-5-R	GAAAGGGGGATGTGCTGCAA	

rhlI-F-XhoI	CCGCTCGAGATGATCGAATTGCTCTCTGAATCG	Forward primer to clone *rhlI* into pBBR1MCS-5
rhlI-R-XbaI	GCTCTAGATCACACCGCCATCGACAGC	
gyrB-RT-F	ACCAGGCCATGGAAGAGTAC	
gyrB-RT-R	CGTTGACCAGTTTCTCCAGC	
rhlI-RT-F	TATTCTGGTCCAGCCTGCAA	RT-qPCR primer
rhlI-RT-R	TCTCGCCCTTGACCTTCTG	
rhlB-RT-F	CATCGCTCACGAGAAGTACGG	
rhlB-RT-R	TCTGCTCGGGCACGTTGAA	
lasA-RT-F	CCGCTGAATGACGACCTGT	
lasA-RT-R	TGATGCTGTAGTAGCCGCTCC	
phzB2-RT-F	GGTGTGGTCGCTGAAATGC	
phzB2-RT-R	TCGTAGTAGCCCTCGGGATAG	
PA1930-RT-F	CCAGATACTGTCCGCCAACC	
PA1930-RT-R	TTCCAGAAGCGCACGTAGGC	
fabH2-RT-F	GCTTCGGACCTCCATCACCTG	
fabH2-RT-R	ACCTCGGCGATGTTGCTCAC	

Table S2. Strains and Plasmids in This Study.

Strains or plasmids	Relevant characteristics	Source
Strains		
P. aeruginosa	Wild-type，PAO1，Km	Laboratory collection
TG1	Host for *E. coli* Cloning vector	Laboratory collection
BL21(DE3)	Host for expression vector pET28a and pGEX-6P-1	Laboratory collection
S17-1	λ-pir lysogen of S17-1, F-thi pro hsdR hsdM+ [RP4-2 Tc::Mu Km::Tn7]	Laboratory collection
ΔrhlI	*rhlI* gene deleted in PAO1	This study
ΔrhlI（vector）	Mutant*ΔrhlI* harboring the plasmid pBBR1MCS-5	This study
ΔrhlI（*rhlI*）	Mutant*ΔrhlI* harboring the expression construct pBBR1MCS-5-*rhlI*	This study
rhlI^{D35A}	Mutant *rhlI*^{D35A} harboring the expression construct PAO1	This study
rhlIK164A	Mutant *rhlI*K164A harboring the expression construct PAO1	This study
Plasmids		
pET28a	Expression vector with His-tag to purify proteins , Km^r	Laboratory collection
pET28a-*rhlI*	pET28a with *rhlI* coding region, Kmr	This study
pET28a-*rhlI*^{D35A}	pET28a with *rhlI*^{D35A} coding region, Kmr	This study
pET28a-*rhlI*^{K164A}	pET28a with rhlI^{K164A} coding region, Kmr	This study
pET28a-*rhlI*^{D35AK164A}	pET28a with *rhlI*^{D35AK164A} coding region, Kmr	This study
pGEX-6P-1	Expression vector with GST-tag to purify proteins, Amp^r	Laboratory collection
pGEX-6P-1-*rhlI*	pGEX-6P-1 with *rhlI* coding region, Amp^r	This study
pGEX-6P-1-*rhlI*^{D35A}	pGEX-6P-1 with *rhlI*^{D35A} coding region, Amp^r	This study
pGEX-6P-1-*rhlI*^{K164A}	pGEX-6P-1 with *rhlI*^{K164A} coding region, Amp^r	This study
pK18 mobsac-Δ*rhlI*	Construct used for deletion of *rhlI*	This study

pK18 mobsac-*rhlI*^{D35A}	Mutant *rhlI*^{D35A} in situ backfilling of the *rhlI* gene	This study
pK18mobsac-*rhlI*^{K164A}	Mutant *rhlI*^{K164A} in situ backfilling of the *rhlI* gene	This study
pBBR1MCS-5	Broad-host range vector, lacZα repmob,Gm^r	Laboratory collection
pBBR1MCS-5-*rhlI*	*rhlI* in pBBR1MCS-5	This study
pBBR1MCS-5-PAO1	PAO1 in pBBR1MCS-5	This study

Note：Km: Kanamycin; Gm: Gentamicin; Amp: Ampicillin.

Table S3. Candidate Proteins for Pip Binding were Identified by Comprehensive Analysis Using the Uniprot Database.

Protein	Description	Coverage	Unique Peptides
HtpG	Chaperone protein	62	42
TufA	Elongation factor	81	39
NrdA	Ribonucleoside-diphosphate reductase	39	39
RhlI	Acyl-homoserine-lactone synthase	50	27
PhzB2	Phenazine biosynthesis protein	39	5
PhzC1	Phenazine biosynthesis protein	23	7
PhzD2	Phenazine biosynthesis protein	22	4
PhzE1	Phenazine biosynthesis protein	19	9
LasA	Protease	31	9
LasB	Elastase	3	1
RhlA	3-(3-hydroxydecanoyloxy)decanoate synthase	32	8
RhlB	Rhamnosyltransferase	21	7
FabH	3-oxoacyl-[acyl-carrier-protein] synthase	15	3

Table S4. Selected DEGs related to virulence, energy metabolism and oxidative stress.

Gene category	Name	Log_2 FC	Gene description
virulence factors			
PA3476	*rhlI*	-1.13	autoinducer synthesis protein
PA3479	*rhlA*	-1.3	rhamnosyltransferase
PA3478	*rhlB*	-1.83	rhamnosyltransferase
PA1130	*rhlC*	-1.28	rhamnosyltransferase
PA1871	*lasA*	-1.68	LasA protease precursor
PA3724	*lasB*	-1.50	elastase LasB
PA1900	*phzB2*	-1.51	phenazine biosynthesis protein
PA4213	*phzD1*	-1.36	phenazine biosynthesis protein
PA1903	*phzE2*	-1.25	phenazine biosynthesis protein
fatty acid synthesis			
PA3333	*fabH2*	-1.68	3-oxoacyl-[acyl-carrier-protein] synthase
